# How to Provide Breast Milk for the Preterm Infant and Avoid Symptomatic Cytomegalovirus Infection with Possible Long-Term Sequelae

**DOI:** 10.3390/life12040504

**Published:** 2022-03-30

**Authors:** Bernhard Resch

**Affiliations:** 1 Division of Neonatology, Department of Pediatrics, Medical University of Graz, Auenbruggerplatz 34/2, 8036 Graz, Austria; bernhard.resch@medunigraz.at; Tel.: +43-316-285-81134; 2 Research Unit for Neonatal Infectious Diseases and Epidemiology, Medical University of Graz, Auenbruggerplatz 38/1, 8036 Graz, Austria

**Keywords:** breast milk, cytomegalovirus, infection, preterm infants, pasteurization, freezing, sepsis-like disease, neurodevelopmental outcome, sequelae

## Abstract

Cytomegalovirus (CMV) is able to replicate in the breast milk of lactating mothers and thus the offspring might be affected by mild to severe symptoms of postnatal CMV disease in case of prematurity; not in term infants. Sepsis-like syndrome affects only very low birth infants; and few cases have been reported. The neurodevelopmental long-term outcome of those preterm infants revealed possible subtle deficiencies, but no major neurodevelopmental impairment. Neurodevelopmental sequelae are still in discussion and seem somewhat overestimated after careful evaluation of the published evidence. The main focus of postnatal CMV disease lies upon the extremely low birth weight of infants. Elimination of CMV is provided by short-term heating methods like the most widely used Holder pasteurization. Freezing and thawing methods leave a risk for CMV acquisition. The benefits of untreated breast milk have to be considered to outweigh the possible sequelae of postnatal CMV infection in the most vulnerable preterm infants.

## 1. Introduction

There is evidence of symptomatic cytomegalovirus (CMV) infection by viral replication during lactation of a CMV seropositive mother to the preterm neonate [1]. There is additional evidence that viral DNA has been detected from colostrum within the first days of life, and detection rates range between 20 and 58 percent [2,3]. Short-term morbidities associated with postnatal CMV infection include a wide range of symptoms, signs and diagnoses like sepsis-like syndrome (the most severe form), hepatopathia, hepatosplenomegaly, thrombozytopenia, neutropenia, hepatitis, myoclony, petechiae, respiratory distress syndrome, hyperbilirubinemia, bradycardia, apneas, cholestasis, distended belly, gray skin color, and elevated liver enzymes [4]. Long-term consequences of postnatal CMV infection still are in debate. For the term neonate, symptomatic CMV disease following postnatal infection has not been described, and is postulated as being a kind of natural immunization [5]. 

## 2. The Rationale for Feeding Human Milk to the Preterm Infant

Breast milk is thought to be the ideal source of feeding for all infants at least for the first six months of life. It provides short and long-term advantages to the infant and its composition varies dependent on maternal genetics, duration of breastfeeding, and maternal diet. Other factors that influence its content include the method of breast milk expression, handling, and storage [6]. In the case of very preterm infants, maternal milk production is frequently delayed or insufficient during the first days after birth. If mother’s milk is therefore unavailable to meet the infants’ nutritional requirements, the best alternative is to use donor human milk [7]. 

### 2.1. Human Breast Milk and the Preterm Infant

Human breast milk produced by mothers of preterm infants is somewhat different in composition compared to milk from mothers of term infants. The milk has higher contents of protein and minerals and contains different types of fat to better meet the needs for a preterm born neonate for at least the first weeks of life. Human milk is easier to digest for the preterm infant and is important for the premature brain and neurodevelopment. Other advantages of human milk are the anti-infective properties including high concentrations of antibodies, lysozyme, and lactoferrin. Hence, breastfed preterm infants are less likely to be prone to intestinal infections than formula-fed neonates [8]. In contrast, the formula-fed preterm infants’ immature intestinal tract is exposed to the cow’s milk protein that increases the risk to develop necrotizing enterocolitis (NEC). Therefore, it is important for the mother to initiate from the beginning to express breast milk to ensure milk supply. In order to have an adequate milk supply, early initiation of frequent and efficient milk expression has been shown to be the most important factor, and an amount of more than 500 mL per day is considered to be significant [9].

Breast milk feeding is the gold standard for every newborn and is sometimes called “white gold” for ill babies and those born preterm. Mothers of preterm infants produce breast milk that is a bit different concerning its composition, at least for the first several weeks compared to mothers of term infants. The premature milk has higher contents of protein and minerals and contains different types of fat that become more easily digested and absorbed. Preterm infants who are breastfed have lower rates of intestinal infections than have babies who are formula-fed, mainly due to anti-infectious properties of breast milk. Human breast milk contains several anti-infective substances that include immunoglobulins, antimicrobial enzymes, and various leukocytes, polyunsaturated long-chain fatty acids, platelet-activating factors, acetylhydrolase, and interleukin-10. In addition, human milk contains some agonists and antagonists of the innate immune responses like CD-14. Together with other factors, they modulate toll-like receptor signaling [10]. As a glycophosphatidylinositol-anchored protein, CD-14 is a co-receptor with toll-like receptor 4 and others for the recognition of lipopoly-saccharides [11].

The benefits of breastfeeding include short, medium and long-term effects, as there is a highly protective effect on infant mortality, a decrease in respiratory and gastrointestinal infections during the first weeks of life of the newborn, and an improvement of neurodevelopmental outcomes both in the term but especially in the premature born population in a quantitative way [8]. Maturation of the gut is given regarding digestive functions but in terms of motility there are lots of problems possibly leading to meconium-obstruction syndrome of the extremely low gestational age newborn. Breast milk and pooled human milk exhibit the advantages of being well tolerated by the premature gut and bear a lower risk of NEC in the very preterm infant. Early and adequate nutritional support is needed to achieve appropriate rates of weight gain, which are almost twice that of a term infant. While early initiation of enteral feedings is beneficial for preterm infants, a rapid increase in volumes of enteral feedings might result in feeding intolerance or NEC [12]. It is important to choose feeding practices associated with improved outcomes for premature infants, thus, breast milk or donor breast milk are the preferred sources of preterm babies’ nutrition, especially during the first weeks of life.

Clinical benefits of early feeding in premature infants have been demonstrated in several trials [13,14,15,16,17,18]. These include better feeding tolerance, more rapid maturation of intestinal motility patterns, increased lactase activity, higher serum levels of gastrointestinal hormones, reduced intestinal permeability, decreased risk of late-onset sepsis, lower incidence of conjugated hyperbilirubinemia, and greater absorption of calcium and phosphorus, resulting in lower rates of osteopenia of the preterm infant [19,20,21].

### 2.2. Donor Human Milk and Milk Banking

Human milk banks play an important role in the nutritional management of very preterm infants who would otherwise be prone to cow milk protein during the vulnerable phase of the first week of life. Milk banks collect, screen, store, process, and distribute human milk from lactating women who produce more breast milk than their own infants need [22]. Donor women are therefore carefully screened for perinatal infectious diseases (HIV-1, HIV-2, human T-cell leukemia virus 1 and 2, hepatitis B, hepatitis C, and syphilis) and for relevant medications. Handling, storing, processing, pooling, and bacterial screening of donor milk follow standardized algorithms, and pasteurization remains standard treatment [22]. Despite the negative influence of pasteurization on the anti-infective properties of donor milk, including cellular components, growth factors, and nutrients, the beneficial effects of donor milk outweigh these disadvantages. According to Picaud [23], donor human milk is the ideal feed in case of delays of successful breastfeeding in hospitalized neonates. Importantly, donor human milk does not negatively affect the use of the mother’s own milk and the rates of breastfeeding at discharge. Donor human milk helps to reduce the rate of typical complications of prematurity like NEC. When it is adequately fortified, the results are depicted in regular postnatal growth charts of the majority of very preterm infants. It should be the priority of the management that is responsible for a pediatric-neonatal unit to establish well-organized and accessible human milk banks covering the needs of very preterm infants, those with gut malformations needing surgical treatment, and those with hemodynamically significant congenital heart disease.

Arslanoglu et al. [24] summarized the evidence for benefits of donor human milk compared to preterm and term formula. Despite different comparisons–formula versus unfortified donor human milk, preterm formula versus fortified donor human milk–and pasteurization not in all studies, the rate of NEC was significantly higher in the formula fed groups with a 2.5-fold increased relative risk. As suggested by pooled estimate, one extra case of NEC out of 33 preterm infants will occur in formula fed infants [25], and further meta-analyses confirmed these findings [26,27].

## 3. Postnatal Cytomegalovirus Infection

Cytomegalovirus (CMV) belongs to the Herpesviridae family and is classified as human herpesvirus type 5. CMV is a common virus for people of all ages provided the immune system is not compromised and prohibits illness. In healthy children and adults, symptoms of CMV infection are usually minimal, mimicking a mild upper respiratory tract infection (fever, sore throat, fatigue, swollen glands), and most of the patients are not aware that they are infected. Over half of adults have been infected with CMV by the age of 40 years; and the virus stays in the body for the whole life and can reactivate, for example during immunosuppressive therapy or in case of malignancies. Re-infection with a different strain of the virus is possible. Most people with CMV infection have no symptoms and aren’t aware that they have been infected. According to the Centers of Disease Control and Prevention, transmission routes include all body fluids, such as saliva, urine, blood, tears, semen, and breast milk [28]. CMV spreads from an infected individual by direct contact with saliva or urine (the most common sources are infants and young children), from breast milk to nursing infants, through sexual contact, and finally through transplanted organs and blood transfusions.

### 3.1. Short-Term Sequelae of Postnatal CMV Infection

Transmission route via breast milk is a kind of natural immunization of term infants, but this is not the case for the preterm infant. The sequelae of congenital CMV infection are well defined: They include microcephaly, seizure disorders, cognitive disability, developmental delay, and sensorineural hearing loss (SNHL) [29]. In contrast, sequelae of postnatal CMV infection via breast milk in the preterm infant is much less clear. Short-term morbidities including sepsis-like-syndrome and diverse end-organ disease manifestations are well recognized. However, the risks of adverse neurodevelopmental outcome are still the subject of debate and discussion. Interestingly, about 90 percent of preterm infants of seropositive mothers acquire CMV via lactation and breast feeding, but only a minority of infants develop signs and symptoms of CMV infection [4,29]. There exist many studies that endeavour to understand this phenomenon. Additionally, factors that modulate reactivation and cessation of viral shedding are the focus of research groups including cytokines, lactoferrin, and CD8+ T cells phenotypes [5]. Transmission of protective maternal antibodies starts at about the 29th week of gestation. This might be the reason why full term infants in general do not develop symptomatic disease [29]. Other manifestations of postnatal CMV have rarely been reported, including NEC [30].

In a study of infants below 1500 g birth weight, venous blood samples were tested for CMV IgG and IgM antibodies on the fifth and 30th day after birth from both mothers and infants (*n* = 38 and 42, respectively) [3]. Breast milk CMV DNA detection by PCR and viral cultures were done until 12 weeks of age, as were urine samples of the infants for CMV culture. Thirty-six mothers (97.3%) were CMV-seropositive, and six infants became infected at a mean age of 77 days after birth. These infants more frequently had sepsis-like syndrome and direct hyperbilirubinemia, but neurodevelopmental outcomes at six months corrected for premature age did not differ between infected and non-infected infants [3].

More than 10 years ago, we published a review on transmission of human CMV via breast milk to the premature infant. Studies revealed CMV-positivity of the infants from CMV-IgG positive mothers from 5.7 to 58.6 percent; symptomatic CMV disease occurred in a median of 3.7 percent of the infants (range 0–34.5%), and severe sepsis-like syndrome in a median of 0.7% (range 0–13.8%) [4]. Few studies reported on long-term sequelae, and only weak evidence exists of mild neurologic and cognitive impairment without hearing impairment. Hamele et al. [31] reported on five preterm infants of 24 (+5) to 27 (+1) weeks of gestational age exhibiting severe morbidity and mortality associated with postnatal breast milk-acquired CMV infection. Since the early 1970s, eighteen infants had been identified when human breast milk first was known to be a potential source of CMV infection. In two cases out of these eighteen infants, the authors provided no further details; five cases, with a gestational age of 29 to 33 weeks, did not experience severe disease (as defined as sepsis-like syndrome). The remaining 11 infants (four studies) had gestational ages ranging from 23 to 28 weeks (23, 25, 24 to 28, and 24.4 ± 0.5 weeks, respectively). 

A Danish study included 26 preterm infants who received their mothers’ own milk and looked at the frequency of CMV transmission, association with viral loads, and rates of sepsis-like symptoms [32]. Despite being a small study, nevertheless, four infants acquired CMV infection, with two of them exhibiting sepsis-like symptoms. The main finding was a higher viral load of mothers’own milk in infected compared to uninfected infants. Thus, viral loads and the amount of mothers’milk significantly increased the risk of CMV transmission to the preterm infant.

### 3.2. Potential Adverse Long-Term Neurodevelopmental of Postnatal CMV Infection

As already stated 10 years ago [4], there still exist very few studies addressing the long-term outcomes of preterm infants having had symptomatic postnatal CMV infection acquired via breast milk. From those studies available, the information is not conclusive. Thus, the principle question whether there remain neurodevelopmental sequelae or not is not answered simply by a “yes” or “no”. Focusing on those studies dealing with breast milk-acquired CMV infection revealed five studies reporting long-term follow-up data of preterm infants with postnatal CMV infection [4]. We found no association with sensorineural hearing loss and no differences regarding motor or speech development in comparison to reported controls. One small study, which found no differences in detailed examinations between postnatally infected and matched controls, reported that even having had severe sepsis-like syndrome, the risk was very low to detect neurologic or cognitive sequelae or to find an increased risk for hearing impairment [33].

Later studies, again from the Tübingen group of Hamprecht and colleagues, looked at more subtle deficits. Of 41 infants investigated at school age, all had normal hearing function and neurodevelopmental testing with the Kaufmann ABC test, and this did not differ between groups [33]. Further analysis of these patients revealed lower results in the simultaneous processing scales of the Kaufmann ABC tests [34]. Since these findings represent more complex cognitive function, they could be of some major concern for the individual. Another follow-up study of 42 infants found lower results in the simultaneous processing scales, even after considering socioeconomic status. Results for the sequential and achievement scales of the Kaufmann ABC were marginally reduced [35]. At adolescent age, a study with 42 former preterm infants compared the infants with term neonates using the Wechsler Intelligence Scale and the Developmental Test for Visual Perception. They scored significantly lower compared with the full-term neonates and had lower scores on overall cognitive abilities compared with the group of preterm infants without CMV infection [36].

Additionally, functional MRI examination showed differences in gray matter volume in several regions while performing different tasks. Hence, either activation differences were observed in the left hippocampus and the right anterior cingulate cortex during language tasks or within a small region of the occipital cortex during visuospatial tasks [37].

Looking at neonates who failed newborn hearing screening revealed a higher rate of 16% in the group with postnatal CMV infection compared with 9% in controls [38]. This study from the Pediatrix Medical Group network included more than 75,000 infants, with 273 infants having had postnatal CMV infection. The conclusion of this study is the fact that comprehensive audiological and neurodevelopmental follow-up data are needed in order to answer the questions regarding possible sequelae of postnatal CMV infection.

### 3.3. Are All Preterm Infants Prone to the Risk of Symptomatic CMV Disease?

As Alan Jobe [39] stated, severe cases of CMV pneumonia or sepsis-like syndrome can occur, but the small number of cases identified in this series is not enough to really estimate the general risk. It is still an unresolved problem as to whether the published evidence of the benefits of feeding preterm infants fresh CMV positive milk is worth the possible acquisition of symptomatic CMV disease by some of the infants. Until 2010 [40] we calculated only few cases of breast milk-acquired symptomatic CMV infection from the literature presenting as sepsis-like syndrome [3,31,41,42,43,44,45]. The gestational age (as shown above) was a maximum of 28 weeks. Therefore, it seems difficult to draw conclusions from such a small cohort of infants and generalize recommendations to all preterm infants. The majority of reported cases with severe postnatal CMV infection are the extremely low gestational age neonates.

## 4. Preventing Postnatal CMV Infection

Regarding prevention of breast milk acquired CMV infection, the only secure method of eliminating virus infectivity is heat inactivation, and short-term heat inactivation is most preferable due to its less detrimental effects on the anti-infective properties of breast milk, and, worthy of note, this short-term heat inactivation can effectively be used under routine conditions. Short-term heat inactivation for 5 min at 62 °C combines the benefits of feeding breast milk without the disadvantages of CMV transmission.

Holder pasteurization (62.5 °C for 30 min) and short term heating (72 °C for 5 s) as well as boiling (5 min) reduce CMV below detectable levels [46]. Pasteurization is the preferred procedure to inactivate CMV in breast milk, since freezing does not completely eliminate the virus. Neither freezing breast milk at up to −20 °C nor storage at 4 °C for different durations (up to one year) are capable of eliminating CMV completely, but concentrations of anti-infective properties of breast milk are barely altered by freezing in contrast to heating procedures [47].

Holder pasteurization at 62.5 °C for 30 min is the most commonly used treatment in human milk banks to eliminate CMV and to ensure its microbiological safety. Nevertheless, these heating methods have a detrimental impact on some of the nutritional and bioactive qualities of human milk [48]. Human milk carbohydrates and lipids appear to be unaffected by Holder pasteurization [49,50], but the concentrations of proteins like lactoferrin and lysozyme [51] or the activity of milk triglycerides digesting bile salt-stimulated lipase get reduced or lost after this procedure [52]. Additionally, some vitamins such as vitamin C, folate or B6 were found to be lower after Holder pasteurization; and others like vitamins A, D and E seemed to be unaffected [48,53]. Recently, high-temperature short-time pasteurization (72 °C for 15 s) has been proposed as an alternative treatment for donor breast milk [54,55,56]. Escuder-Vieco et al. [57,58] created a continuous high temperature short time system that was developed to pasteurize donor breast milk in the milk banking-operating environment. It was designed to ensure microbiological safety by preservation of some of the bioactive factors. Further studies from this research group revealed that the duration of this procedure had a greater influence on the qualitative composition of donor breast milk than the temperature [59]. Escuder-Vieco et al. concluded that high temperature short-time pasteurization resulted in better preservation of the nutritional quality of donor breast milk than Holder pasteurization. Microwave radiation (e.g., 500 W for 40 s) has been introduced successfully, but we do not know the side effects regarding the loss of the bioactive properties of breast milk [60,61].

## 5. Discussion

Pasteurization is clearly the preferred procedure to inactivate CMV in breast milk, since freezing is not the method that eliminates the virus completely [47]. In order to be at the safer side one has to decide on a heating method that guarantees CMV elimination and prohibits postnatal infection. However, heating procedures significantly reduce protective factors covered by breast milk, which are elemental for the benefits and positive long-term effects guaranteed by breastfeeding [62]. Despite the fact that severe CMV disease happens in a minority of very low birth weight infants, an individual decision based on the health status of the preterm infant instead of a general approach by either pasteurization or the withholding of breast milk would be preferred.

**Figure 1:**
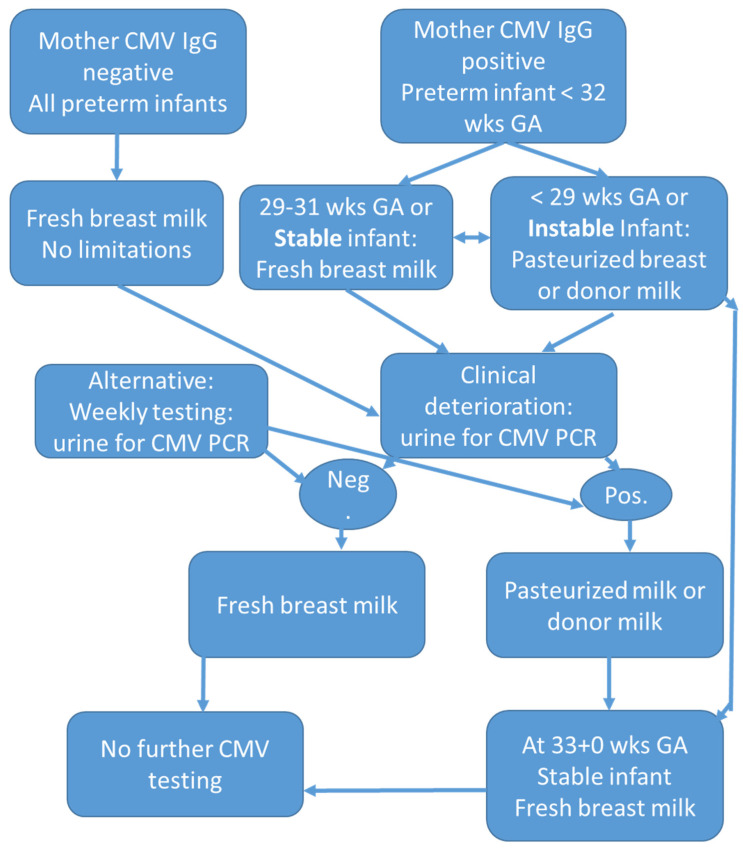
Algorithm for providing fresh or pasteurized breast milk to preterm infants below 32 weeks of gestational age dependent on their postmenstrual age. In case of maternal negative status regarding cytomegalovirus (CMV) infection, fresh breast milk might be provided to all preterm infants. In the case of mother being CMV IgG positive and a 28 weeks’ gestation neonate or younger, pasteurization might be justified that can be stopped at 33 + 0 weeks postmenstrual age.

Therefore, parents need to receive information about the risk of CMV disease when feeding their preterm infant with fresh breast milk and the benefits of feeding untreated breast milk. Provided parents give informed consent and the preterm infant is in stable condition, then fresh breast milk can be used (see Figure 1). The suggested algorithm includes a careful evaluation of the risks and benefits of untreated breast milk related to the infants´ current health status. Weekly CMV monitoring in the infant by PCR from urine samples is an alternative to the wait and see strategy at the right side of the decision tree. If the infant tests positive, feeding them fresh milk should be stopped. Thus, viral loads will not further aggravate symptoms and signs of CMV infection [41]. Otherwise, if theinfant develops no symptoms or signs of CMV infection, feeding them fresh breast milk can be continued provided the gestational age is above 28 weeks. A postmenstrual age of more than 32 weeks might justify feeding fresh breast milk even in the tiniest preterm infants. I still share the view of Stagno et al. [63] that the well-established value of breastfeeding clearly outweighs the low risk associated with the transmission of CMV through breast milk. Passive immunization with either CMV monoclonal antibodies or immune globulins might be discussed for high-risk preterm infants but seems to be a more theoretical procedure. For these babies, pasteurized milk is the preferred strategy.

The Red Book Committee of the American Academy of Pediatrics recommends serological CMV screening for mothers of preterm infants born at less than 32 weeks of gestational age, and for those being CMV-seropositive it recommends the short-term pasteurization of breast milk [64]. This is, to my point of view, not justified for all those preterm infants considering the low risk of severe CMV infection and the uncertainty regarding long-term neurodevelopmental sequelae. The Committee also considers the freezing of breast milk at −20 °C for 24 to 72 h as not being able to eliminate the risk of infection [64,65]. A study by Omarsdottir et al. [66] additionally failed to demonstrate any benefit of freezing breastmilk on CMV transmission in preterm infants of less than 28 weeks of gestational age.

The information regarding long-term outcomes following breast-milk acquired CMV infection still is inconclusive. Only a small percentage of infants exhibit symptoms and signs of CMV central nervous system disease; and deficits described are based on mild cognitive deficits. In a study by Brecht et al. [36], there was a considerable divergence between the CMV-infected and those without postnatal infection regarding IQ (98 vs. 110) and on visuoperceptive abilities (96 vs. 106). Preterm infants with early postnatal CMV infection scored significantly lower than those without this infection regarding overall cognitive abilities (93 vs. 103), but not in terms of visuo-perceptive abilities. Of immense importance seems to be the question of whether early-life acquisition of CMV from breast milk in VLBW infants confers an increased risk of SNHL. In a follow-up study of very low birth weight preterm infants, no evidence of SNHL was noted when infants were evaluated at 2.0 to 4.5 years of age [36]. Another long-term follow-up study of premature infants using several developmental test batteries demonstrated no effect of CMV on neurodevelopment [67]. A very recent study included 304 infants with postnatal CMV from a total of 75,000 infants from 302 NICUs screened between 2002 and 2016, and 273 (89.8%) of these were matched to control infants without postnatal CMV infection [38]. Newborn hearing screen failure occurred in 16.5% of cases with postnatal CMV compared with 9.2% without postnatal CMV. The authors additionally observed an increased postnatal age at discharge (about 12 days) and a lower weight-for-age z score. Thus, they concluded that CMV infection led to prolonged hospitalization and was negatively associated with growth. Limitations of this study include the low rate of postnatal CMV infection (0.4%) and the fact that a failed hearing screen did not imply SNHL [68].

## 6. Conclusions

CMV infection should be considered in very low birth weight infants who are breastfed by seropositive mothers and presenting severe or sepsis-like symptoms with negative cultures [69]. To prevent the vulnerable tiny preterm infants from breast milk-acquired CMV infection, only heat inactivation eliminates virus infectivity, and short-term heat inactivation is most preservative. Short-term heat inactivation for 5 s at 62 C maintains the benefits of feeding breast milk without the disadvantages of CMV transmission [70]. Further large prospective trials are needed to resolve the question of the neurodevelopmental implications of breast milk-acquired CMV in the very low birth weight infant, and even more knowledge is needed regarding the mechanisms of the underlying biology. In the meanwhile, pasteurization is the only method to eliminate CMV and, thus, to prohibit infection of the very preterm infant, but the risk defers mainly to the most vulnerable and premature babies.

## Data Availability

Not applicable.
